# Orthopedic infections: no one is denying anymore that we have a problem!

**DOI:** 10.1186/s43019-019-0026-y

**Published:** 2019-12-24

**Authors:** Javad Parvizi

**Affiliations:** James Edwards Professor of Orthopedic Surgery, Sidney Kimmel Medical College, Rothman Institute at Thomas Jefferson University Hospital, Sheridan Building, Suite 1000, 125 South 9th Street, Philadelphia, PA 19107 USA

My first encounter with orthopedic infection was as a second-year resident when a 72- year-old patient with an intractable periprosthetic joint infection (PJI) was facing amputation of her extremity. She declared, “I rather die than lose my extremity.” That fate befell her in the coming year.

Over the last two decades we have made some strides in the field so that not as many of our patients have to face that ill-disposed choice in their care. We have seen young and established investigators engage in basic and clinical research in the field of orthopedic infections. The field that was roamed by a few now houses many intellectuals and innovators who have invested their intellect to address a pressing issue.

Among the many accomplishments has been the identification of the scale of the problem in that orthopedic infections pose a colossal burden on the healthcare system [1]. The recognition of the fact that orthopedic infections may behave like cancer and share many parallels has engendered interest in the issue [2]. The patients with orthopedic infections, like cancer patients, can suffer prolonged hospitalizations, require extensive period of pharmaceutical treatments, stand to lose function, and may perish in the process.

The milestones in research included the introduction of the definition of PJI by societies like the Musculoskeletal Infection Society (MSIS) and the International Consensus Meeting (ICM) [3]. This standard definition allowed us to “sing off the same hymnbook” and standardize other aspects of care. In 2018, the definition for PJI was elevated further when an evidence-based, validated scoring system was introduced that also takes into account the role of novel synovial and serum biomarkers [4].

The introduction of various biomarkers in recent years has contributed immensely to our understanding of orthopedic infections that may masquerade as painful joints with no other clinical or radiographic signs. We have come to realize that other conditions, such as inflammatory arthropathies and adverse local tissue reactions, may mimic PJI, posing challenge for clinicians treating these patients.

One of the main challenges that still lie ahead is the detection of infective organisms that lie in biofilm and may not be detected by routine culture, so-called culture-negative cases [5]. The molecular techniques, such as next-generation sequencing, despite their issues, may be providing us the opportunity to identify the microorganisms causing the orthopedic infections [6].

The recognition of the fact that microorganisms live in a biofilm and may also take refuge inside osteoblasts and other cells has led many investigators to seek biofilm-detection and -disruption technologies. The orthopedic community, riding on the heels of other disciplines, has become aware of the influence of immune-enhancing strategies that may pave the road in the future for better treatment of patients who fall victim to infection. We have also come to recognize the importance of the microbiome in causing and accentuating infective conditions in humans.

Despite all our accomplishments so far, we have a long road to travel. Many challenges lie ahead and we are in desperate need of innovations to change our current practice which appears to be failing our patients. It is not acceptable that PJI carries a 72% mortality at 5 years, akin to many common cancers [1].

The future must be different. It needs to start by us, the orthopedic community, questioning the many misconceptions handed to us, and the routine practices that stand on no scientific footing. The knee-jerk reflexes such as 6 weeks of intravenously administered antibiotics followed by 2 weeks of a “drug holiday” for everyone with PJI is perhaps a primitive and non-scientific approach to the problem. Could it be that the time for individualized medicine taking advantage of molecular techniques, the microbiome, immune-enhancing strategies, artificial intelligence and machine learning is here?
